# Supplementary material for: Characterizing the impact of the COVID-19 pandemic on HIV testing among Medicaid beneficiaries
Source: medRxiv. 2026 Feb 14:2026.02.12.26346199. Preprint. [Version 1] doi: 10.64898/2026.02.12.26346199 (PMC12919114; doi:10.64898/2026.02.12.26346199)
Supplement: Supplement 1 [file media-1.pdf]

Supplemental tables and figures

Supplementary Table 1. List of codes used in the study

Variable	ICD-9 code[a]	ICD-10 Code[a]	CPT/HCPCS
**HIV**	042, 079.53, 795.71, V08	B20, B97.35, R75, Z21	
**HIV testing**		Z11.4	86311, 86312, 86314, 86689, 86701, 86702, 86703, 87389, 87390, 87391, 87534, 87535, 87536, 87537, 87538, 87539, G0432, G0433, G0435, G0475, S3645

[a] The ICD-9 codes for HIV diagnosis were used purely for excluding beneficiaries with HIV diagnosis at baseline while the ICD-10 codes were used for exclusion if the diagnosis is on or before baseline and determination of incidence of infection if the diagnosis date is after baseline.

Supplementary Table 2. Distribution of the follow-up time by baseline year

Baseline Year	Median [Interquartile range]
Overall	1.50 [0.57, 3.16]
2016	3.08 [1.08, 5.50]
2017	1.66 [0.58, 4.16]
2018	1.58 [0.58, 3.42]
2019	2.16 [0.58, 2.58]
2020	1.50 [1.25, 1.58]
2021	0.50 [0.33, 0.75]

Supplementary Table 3. Comparison of observed and predicted values of HIV testing, HIV diagnosis, and test positivity during the pandemic period

	Relative effect in % (95% CI)	Posterior tail-area probability p
**HIV Testing**		
Overall	-8.1 (-12.5, -3.4)	0.005
Female		
18 - <35	-5.1 (-9.2, -0.3)	0.021
35 - <50	-8.3 (-13.3, -2.3)	0.012
50 - <65	-11.4 (-21.5, -0.3)	0.026
Male		
18 - <35	-18.1 (-22.3, -13.8)	<0.001
35 - <50	-14.6 (-20.6, -7.3)	0.004
50 - <65	-11.1 (-18.9, -4.5)	0.006
Black, non-Hispanic		
18 - <35	-5.3 (-9.3, -0.3)	0.022
35 - <50	-8.5 (-15.1, -0.5)	0.022
50 - <65	-12.2 (-23.8, -0.5)	0.027
White, non-Hispanic		
18 - <35	-7.3 (-12.4, -0.1)	0.025
35 - <50	-12.2 (-18.3, -3.6)	0.011
50 - <65	-10.4 (-19.6, -2.1)	0.015
COVID-19 restriction		
Least	-4.8 (-12.1, 3.9)	0.074
Less	-8.0 (-12.1, -4.0)	0.004
Most	-9.3 (-15.4, 1.4)	0.032
**HIV infection**		
Overall	2.2 (-5.1, 9.4)	0.201
Female		
18 - <35	12.6 (-11.5, 76.3)	0.189
35 - <49	13.8 (-11.7, 70.0)	0.159
50 - <65	-10.1 (-37.3, 20.7)	0.189
Male		
18 - <35	2.9 (-4.5,10.5)	0.154
35 - <49	-0.7 (-16.2,12.0)	0.483
50 - <65	2.6 (-6.6,14.8)	0.258
Black, non-Hispanic		
18 - <35	4.9 (-2.8,16.3)	0.090
35 - <49	2.4 (-15.2,16.6)	0.306
50 - <65	2.7 (-10.9,16.9)	0.288
White, non-Hispanic		
18 - <35	8.5 (-9.9, 46.0)	0.206
35 - <49	1.3 (-19.2, 30.8)	0.499
50 - <65	3.4 (-10.0,27.4)	0.361
COVID-19 restriction		
Least	1.0 (-15.1,23.1)	0.498
Less	5.1 (-3.5,14.2)	0.080
Most	-1.1 (-18.5,17.0)	0.380
**Test positivity**		
Overall	6.2 (-14.5, 18.8)	0.121
Female	0.3 (-16.8, 21.4)	0.492
Male	19.7 (-95.5, 38.2)	0.193
Black, non-Hispanic	4.0 (-41.3, 26.2)	0.212
White, non-Hispanic	9.0 (-19.5, 31.9)	0.151
COVID-19 restriction		
Least	-7.8 (-54.8, 22.8)	0.421
Less	12.2 (-27.7, 31.2)	0.107
Most	10.4 (-13.2, 30.7)	0.468

Note: CI – Confidence interval

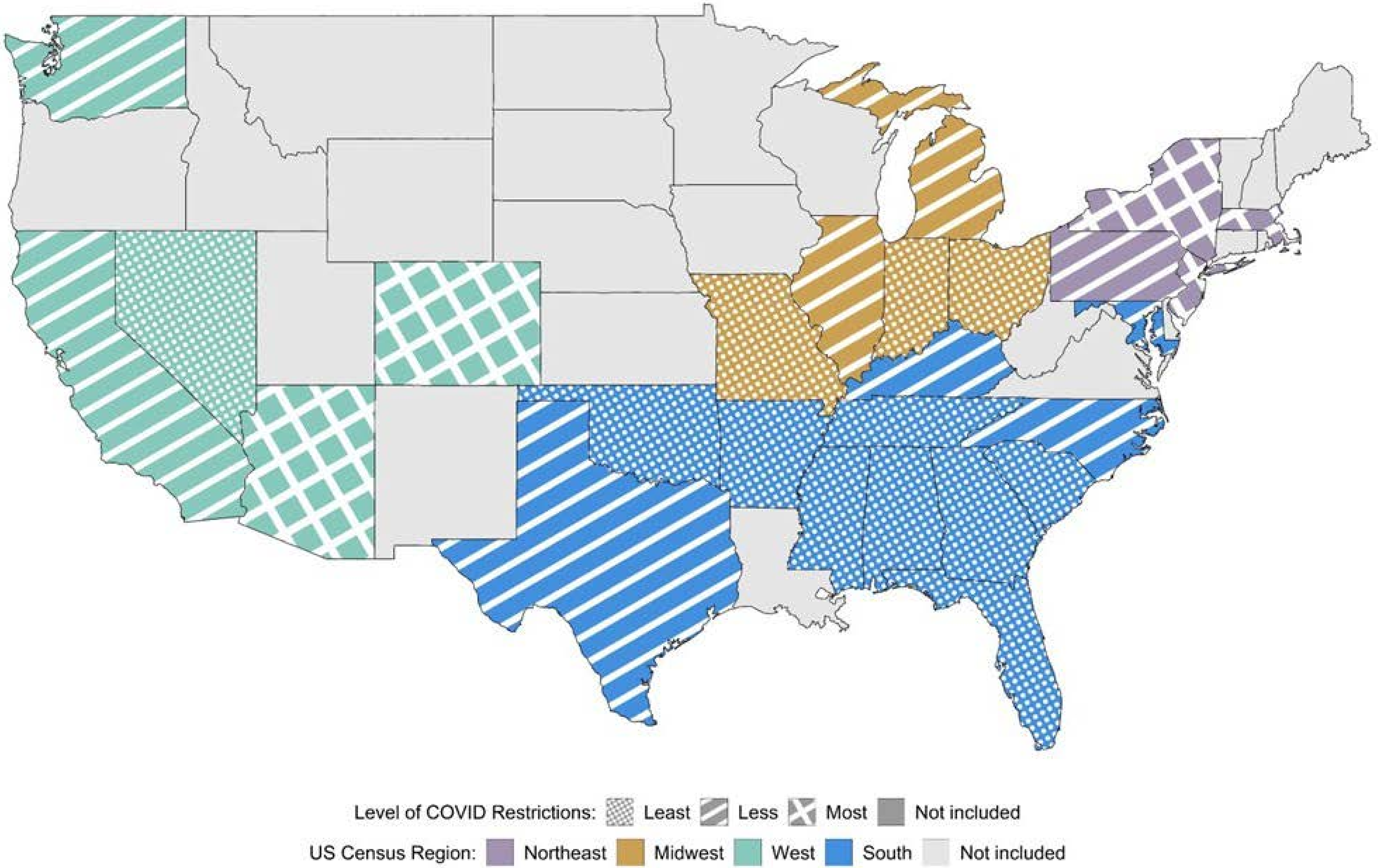

Supplementary Figure 1. US states included in the study by level of COVID-19 restrictions and census region

A

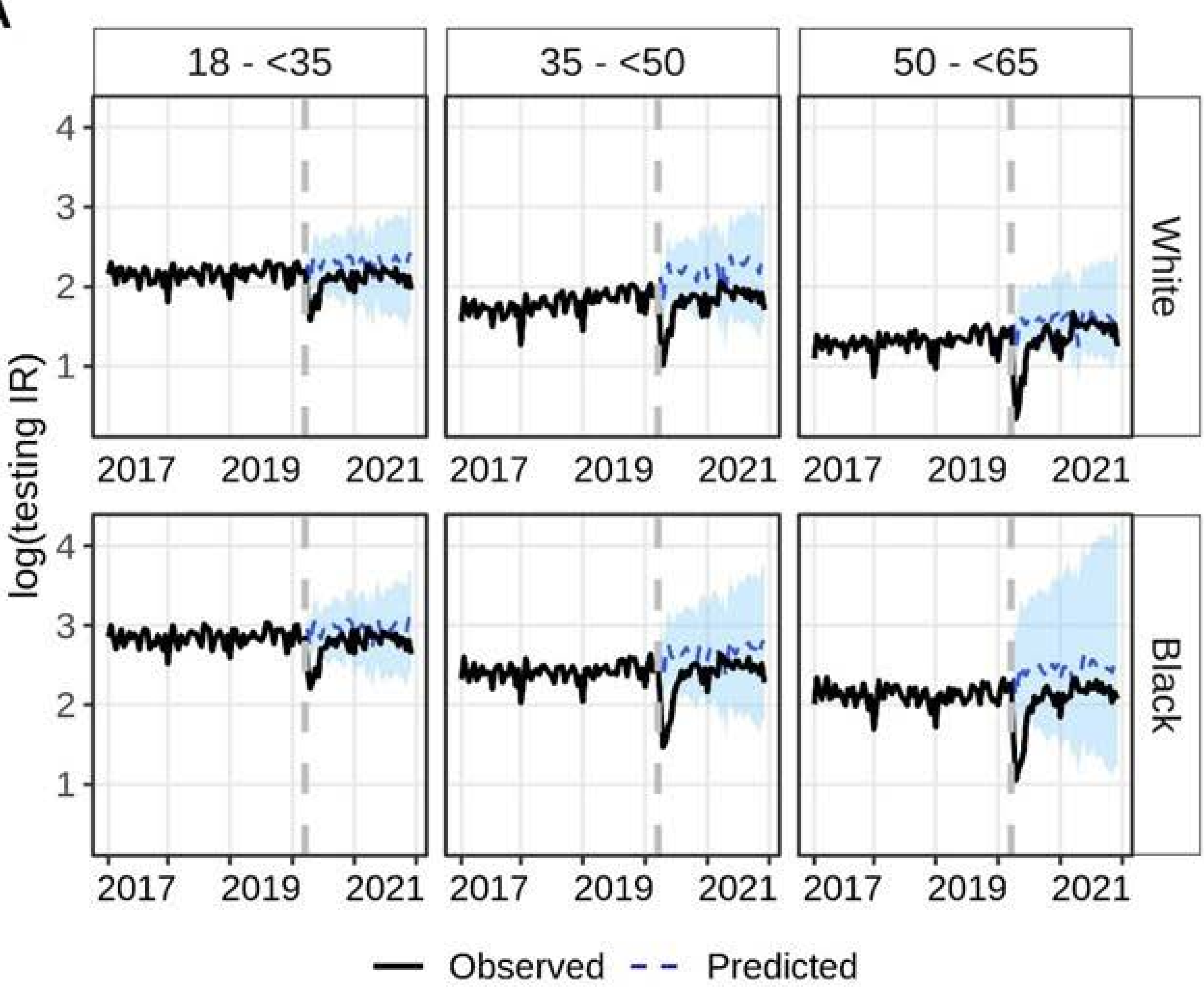

B

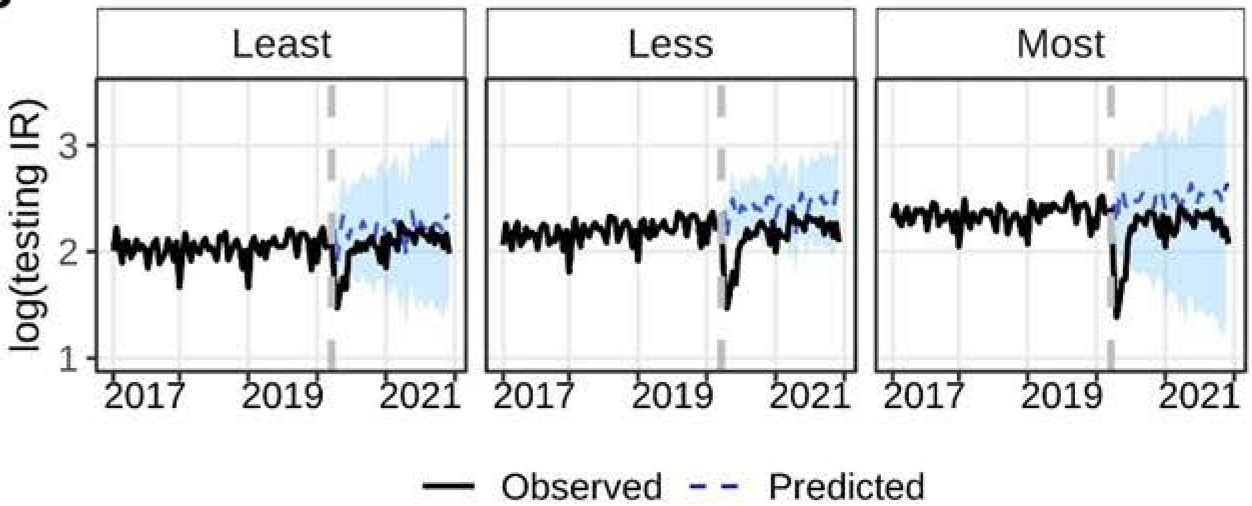

Supplementary Figure 2. Trend of log(standardized HIV testing incidence rate) from January 2017 to December 2021 stratified by A) age and race, and B) states' COVID-19 restriction policies. The vertical gray dashed line separated the pre-pandemic and pandemic periods. The solid lines represent the observed values while the blue dashed lines with blue 95% credible intervals during the pandemic period represent the predicted values had the pandemic not happened.

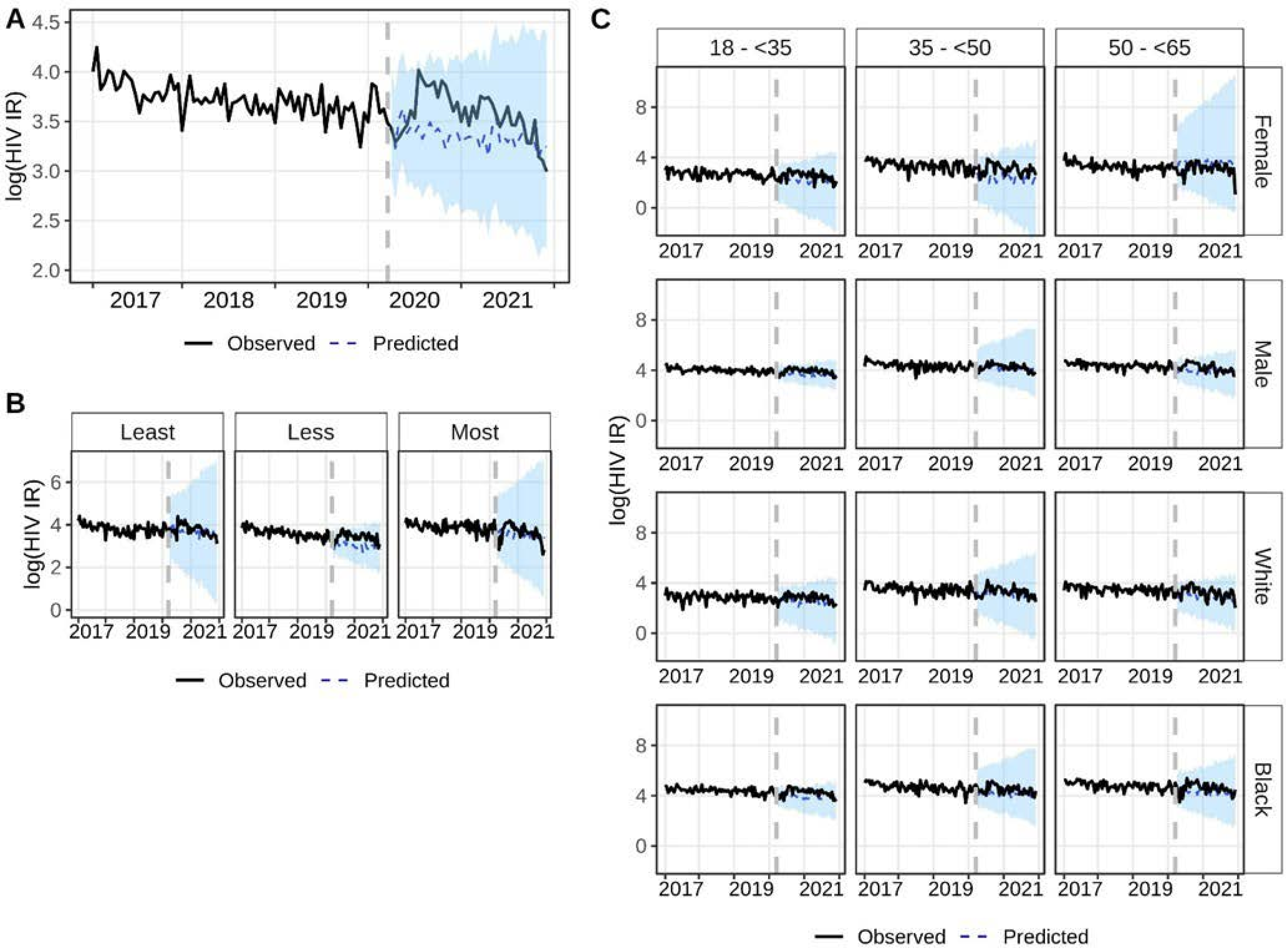

Supplementary Figure 3. Trend of log(standardized HIV incidence rate) from January 2017 to December 2021 A) overall, B) by states' COVID-19 restriction policies, and C) by age, sex, and race/ethnicity. The vertical gray dashed line separated the pre-pandemic and pandemic periods. The solid lines represent the observed values while the blue dashed lines with blue 95% credible intervals during the pandemic period represent the predicted values had the pandemic not happened.

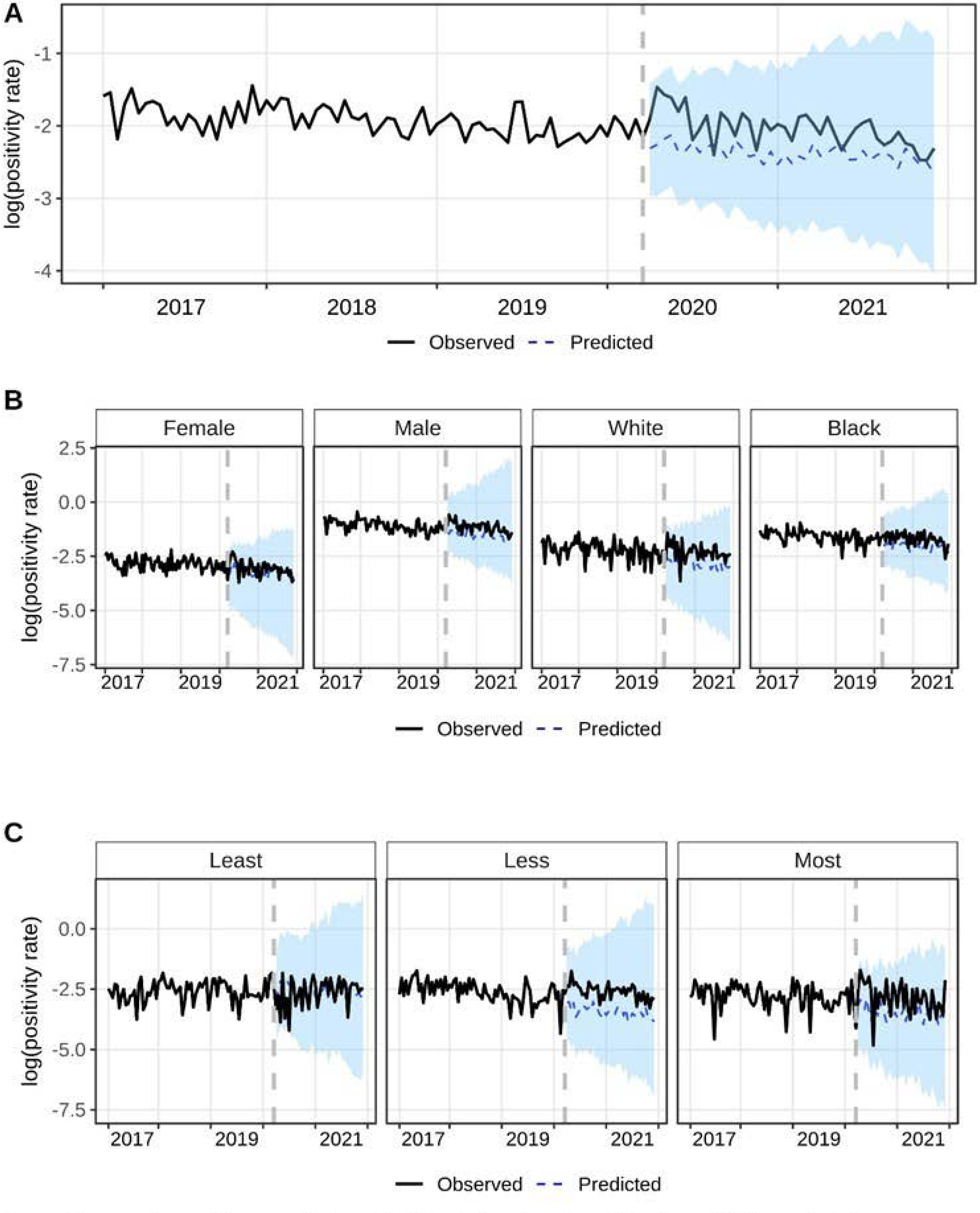

Supplementary Figure 4. Trend of log(standardized test positivity rate) from January 2017 to December 2021 A) overall, B) by sex and by race, and C) by states' COVID-19 restriction policies. The vertical gray dashed line separated the pre-pandemic and pandemic periods. The solid lines represent the observed values while the blue dashed lines with blue 95% credible intervals during the pandemic period represent the predicted values had the pandemic not happened.
